# Accuracy Evaluation of a Stereolithographic Surgical Template for Dental Implant Insertion Using 3D Superimposition Protocol

**DOI:** 10.1155/2017/4292081

**Published:** 2017-05-07

**Authors:** Corina Marilena Cristache, Silviu Gurbanescu

**Affiliations:** ^1^Faculty of Midwifery and Medical Assisting, “Carol Davila” University of Medicine and Pharmacy, Bucharest, Romania; ^2^“Carol Davila” University of Medicine and Pharmacy and Private Practice, Bucharest, Romania

## Abstract

**The aim:**

of this study was to evaluate the accuracy of a stereolithographic template, with sleeve structure incorporated into the design, for computer-guided dental implant insertion in partially edentulous patients.

**Materials and Methods:**

Sixty-five implants were placed in twenty-five consecutive patients with a stereolithographic surgical template. After surgery, digital impression was taken and 3D inaccuracy of implants position at entry point, apex, and angle deviation was measured using an inspection tool software. Mann–Whitney *U* test was used to compare accuracy between maxillary and mandibular surgical guides. A *p* value < .05 was considered significant.

**Results:**

Mean (and standard deviation) of 3D error at the entry point was 0.798 mm (±0.52), at the implant apex it was 1.17 mm (±0.63), and mean angular deviation was 2.34 (±0.85). A statistically significant reduced 3D error was observed at entry point *p* = .037, at implant apex *p* = .008, and also in angular deviation *p* = .030 in mandible when comparing to maxilla.

**Conclusions:**

The surgical template used has proved high accuracy for implant insertion. Within the limitations of the present study, the protocol for comparing a digital file (treatment plan) with postinsertion digital impression may be considered a useful procedure for assessing surgical template accuracy, avoiding radiation exposure, during postoperative CBCT scanning.

## 1. Introduction

Nowadays, cone beam computed tomography (CBCT), advanced technology at reasonable costs and low radiation dose [1, 2], made it possible to better visualize the underlying bone structures for a more precise implant rehabilitation comparing to the standard two-dimensional (2D) radiography.

Proper implant position, “prosthetically driven,” is fundamental in order to achieve an aesthetic and functional implant-supported restoration [3] and can be analyzed and planned with the assistance of numerous types of dedicated software [4].

In order to transfer the planned implant position information to the clinical situation, Jung and coworkers [5] defined two types of techniques: “static,” applying surgical templates, and “dynamic,” transferring the selected implant position to the surgical area via visual imaging tools on a monitor. Dynamic guided implant surgery allows the surgeon to adjust the implant position in real time but is not frequently used, mostly due to the initial high costs of the equipment requested [6].

Static guided implant surgery is preferred due to increased predictability, reduced invasiveness of surgical procedures [7], less healing period required, decreasing treatment time, and increasing patient satisfaction [8].

The accuracy of a guided implant surgery system is defined as the deviation between the planned and placed position of an implant [4].

Implant positioning accuracy is crucial especially when immediate restoration is intended and limited space is available and to avoid damaging the vital structures [9–11].

The protocol of static surgical guidance involves several steps from data collection, to planning, surgical template manufacturing, and effective surgical placement of the implants [12]. Errors can occur at each individual step and the final inaccuracy is the sum of all mistakes.

Assessing the overall errors with a static guided implant protocol is mandatory in order to

- improve the design and manufacturing of the surgical template and the overall protocol of implant insertion,
- plan the implant position at a convenient distance, considering the occurrence of insertion inaccuracy, to elude complications and also to avoid damage of vital structures,
- provide precise prosthetic reconstructions prior to surgery, resulting in reduced treatment time.

The accuracy assessment between planned and placed implant position was, in most of the studies published, based on matching preoperative and postoperative CBCT over the treatment plan [13–16], requiring radiological investigation with higher irradiation dose, not in accordance with ALARA principles.

A method of surgical template accuracy assessment avoiding a second CBCT investigation is needed [17].

Therefore, the aim of this study was to evaluate, by superimposition of 3D digital files, the accuracy of computer-guided dental implant insertion in partially edentulous patients using a stereolithographic template with sleeve structure incorporated into the design.

The null hypotheses of the present study were formulated as follows:

1. Neither angular nor 3D deviations would be found between the planned and placed implant position with the proposed computer-guided surgery protocol.
2. If present, no statistically significant deviations would be found in all directions between maxillary and mandibular implants inserted.

## 2. Materials and Methods

Twenty-five consecutive partially edentulous patients (20 women and 5 men, age ranged between 32 and 66, mean 51 years), included in Classes I and II, according to the American College of Prosthodontists classification [18], requiring dental implant placement, were enrolled in this prospective clinical study (ClinicalTrials.gov Identifier: NCT02418117) conducted, between April 2015 and December 2016, in accordance with ethical principles including the World Medical Association Declaration of Helsinki and approved by the Bioethics Committee of “Carol Davila” University of Medicine and Pharmacy (70/04.06.2015). Written consent of each subject was also obtained.

Patients with limited bone volume requiring staged bone graft, limited mouth opening (impossibility of using a surgical template), or history of Parkinson disease (impossibility of performing an accurate CBCT) were excluded from the present study.

### 2.1. Patient Data Collection

After initial examination, an accurate impression of the surgical site and the opposite arch, for stone casts, was taken to all patients. For perfect 3D matching of the scanned models with the CBCT files, a radiopaque datum tray (R2Tray®, Megagen Implant, Gyeongbuk, Korea) was customized with silicone (Registrado Clear®, VOCO, GmbH, Cuxhaven, Germany) on the dental arch to be restored with implants. Same silicone was utilized for bite registration.

A larger volume CBCT was performed for each patient with the customized datum tray, using ProMax 3D (Planmeca®, Helsinki, Finland) with a rotation of 360°, for data acquisition. All CBCTs were performed with the following characteristics: field of view (FOV: height and diameter) was 160 mm and 160 mm, voxel size was 0.3 mm, and the exposure factors were 110 kV, 6.0 mA, and 13.779 s exposure time, patient's Camper plan (Ala-Tragus) parallel to horizontal plane.

A series of axially sliced image data were obtained and exported to a personal computer in DICOM (Digital Imaging and Communications in Medicine) format. Stone models, individually and in centric occlusion and datum trays, were scanned using a D 700 3D scanner (3Shape®, Copenhagen, Denmark) and imported as stl (standard tessellation language) files.

### 2.2. Treatment Plan

**Figure 1:**
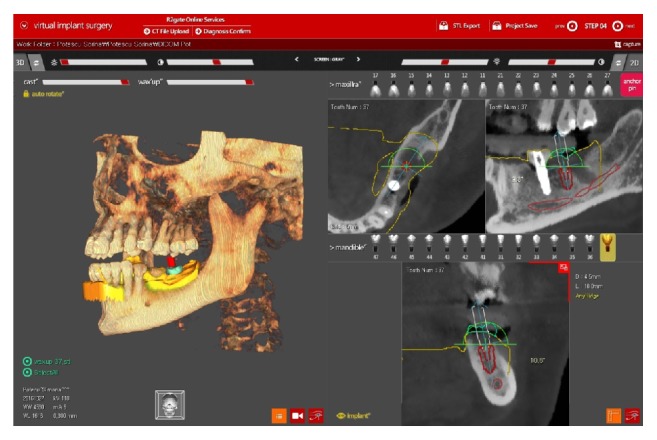
Planned implant insertion in R2GATE® software.

DICOM files obtained from CBCT and stl files were imported in a treatment plan software R2GATE version 1.0.0 (Megagen, Gyeongbuk, Korea) and R2Tray was used as landmark for superimposition of the scanned model and underlying bone image. Implants length and diameter were selected and drilling protocol was planned according to the final restoration and bone anatomy (Figure 1).

**Figure 2:**
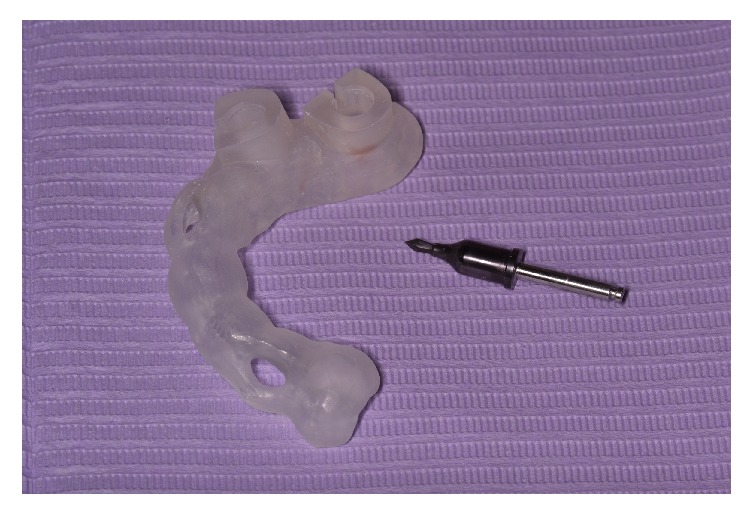
The stereolithographic surgical guide utilized in all cases had the guide sleeve incorporated in the design, eliminating the need for additional insertion of metal guide sleeves. All surgical drills used had 3 parts: the stopper part, the guide part, and the drilling part [19]. Stopper and guide parts are identical for all drills and especially designed for R2Gate® surgical template. Drilling part varies in length and diameter according to the drilling protocol.

A surgical template was designed and fabricated, for each treatment plan, using Clear Guide M, a light curing material to be used in an additive manufacturing technology (Stereolithography) with EnvisonTEC Perfactory®3D printer (Gladbeck, Germany). The surgical template used is sleeve incorporated (Figure 2), requiring shank-modified drills for minimizing mechanical tolerance of the instruments and increasing accuracy, as described by Lee and coworkers [19].

### 2.3. Implant Surgery

All 65 implants inserted were AnyRidge® (Megagen Implant, Gyeongbuk, Korea) and all surgeries were performed according to the manufacturer's instructions, by one experienced surgeon, under local anesthesia, using flapless, minimally invasive technique.

Perfect fit of the template was assessed prior to surgery (on the diagnostic gypsum cast) and intraorally, on adjacent teeth. Adequate mouth opening after surgical template insertion was also verified in order to avoid displacement of the surgical instruments during site preparation.

**Figure 3:**
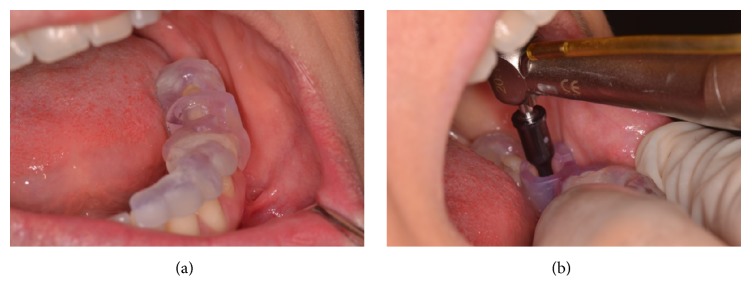
(a) Surgical template applied over the edentulous area and adjacent teeth. (b) Second drill used for flapless implant site preparation.

The tooth-supported surgical template was applied over the edentulous area and adjacent teeth and the corresponding shank-modified drills were used (Figures 3(a) and 3(b)).

**Figure 4:**
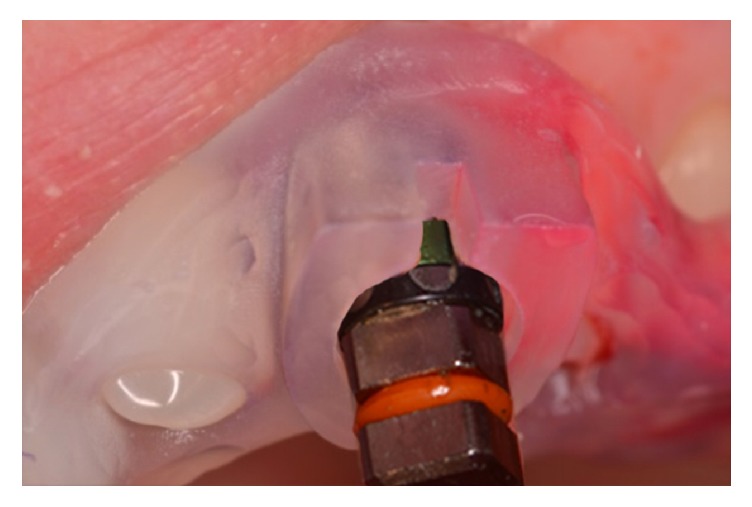
Implant insertion with hand ratchet. The ratchet connector has six green vertical landmarks (corresponding to implant hex) and a horizontal reference line. In order to reproduce the planned implant position, the horizontal reference line should match with the upper border and the green vertical landmark with the window of the surgical template.

A fully guided site preparation and implant insertion was performed. Implants were inserted using a hand ratchet up to the required landmark in order to reproduce the planned insertion depth (Figure 4).

### 2.4. Accuracy Assessment

**Figure 5:**
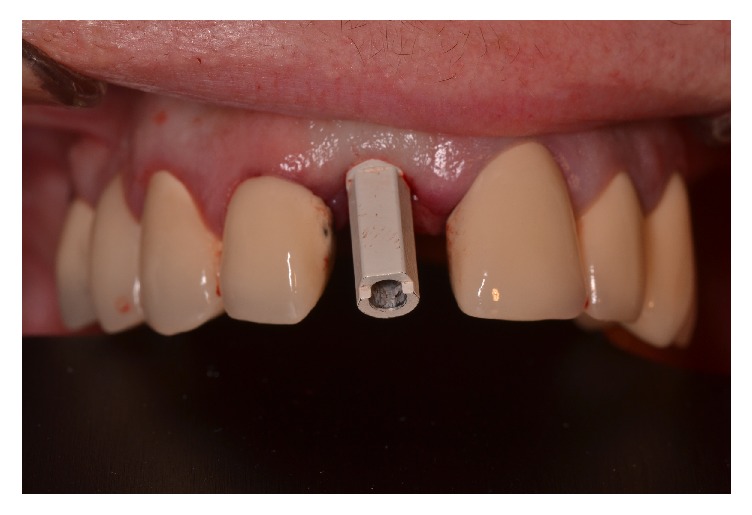
Digital impression of the implants after screwing the scan abutment.

After implant insertion, digital impression was performed using the intraoral scanner CS 3500 (Carestream Health, Inc., Rochester, NY, USA). Standard scan abutment was screwed onto each implant prior to impression (Figure 5) and the obtained stl file was imported in Geomagic Qualify 2013 software (Rock Hill, SC, USA). The stl file of the corresponding inserted implant (length and diameter) was then attached to each implant by perfect matching of the scan abutment, using* best fit algorithm*.

Treatment plan exported from R2GATE software, as stl file as well, with scan abutment included was also imported in Geomagic Qualify 2013 software and the corresponding implant stl file was added for each fixture.

For examining the deviation between the planned and placed position of each implant, treatment plan data set and digital impression with scan abutments were superimposed.

Treatment plan was set as reference, the 3D coordinate axes were defined (*x*: buccolingual, *y*: mesiodistal, and *z*: apicocoronal), and the digital impression was aligned to the reference using the best fit algorithm [20]. Alignment was performed for perfect matching of the neighboring teeth.

To facilitate an accurate evaluation, irrelevant areas, beyond the field of interest, were removed.

**Figure 6:**
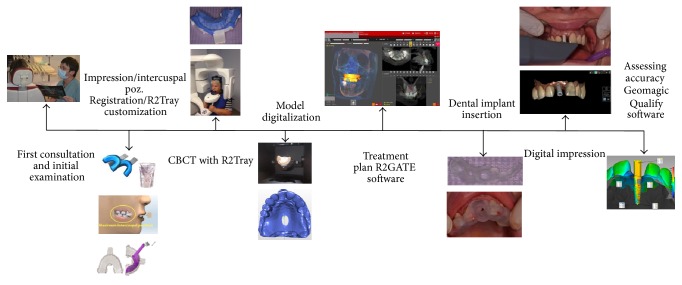
Study workflow.

The entire work flow is presented in Figure 6.

**Figure 7:**
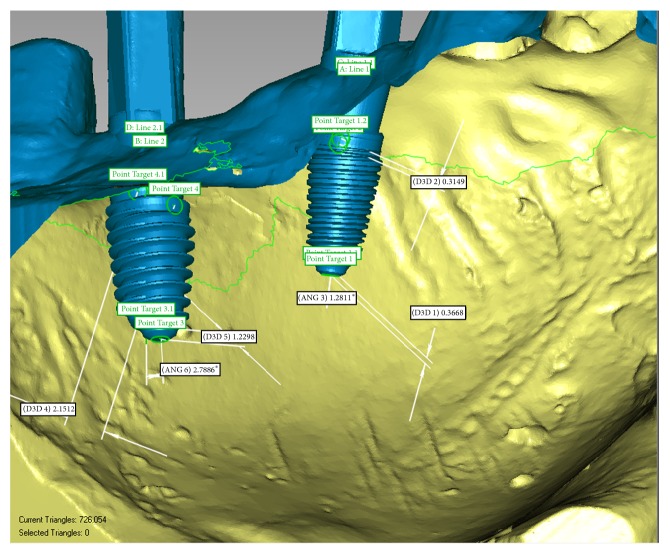
Measurement of 3D accuracy of the planned (reference) and effective implant insertion (test), *∗* represents degree symbol (°) as it measures an angle.

For accuracy analysis the following parameters were assessed [17] using Geomagic Qualify 2013 software (Figure 7):

- 3D error at the entry point measured at the center of the implant (in mm),
- 3D error at the apex measured at the center of the implant apex (in mm),
- angular deviation,
- vertical deviation at entry point measured at the center of the implant (on *z*-axis).

The 3D deviation was calculated by the software taking into consideration the deviation on each direction set as follows: *x* = buccolingual error, *y* = mesiodistal error, and *z* = apicocoronal error, using Pythagorean Theorem [17]:

(1) $$\begin{array}{c} 3D\;\;dev.=\sqrt{x^{2}+y^{2}+z^{2}}. \end{array}$$

**Figure 8:**
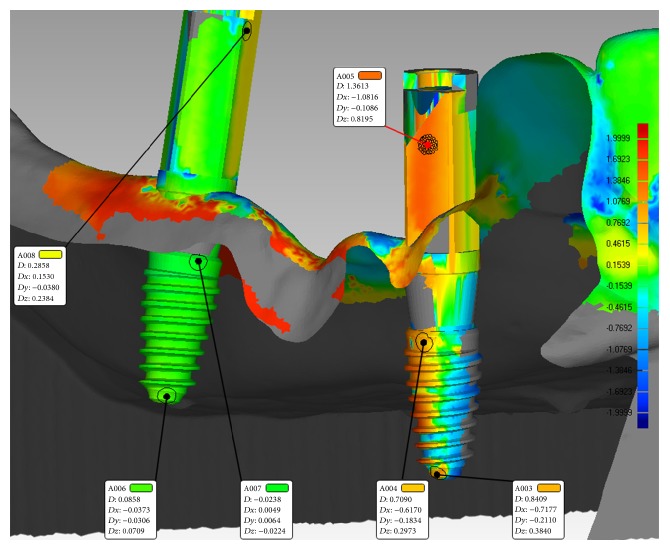
Qualitative color-codded graphical analysis of implants planned (reference) and placed (test) in Geomagic Qualify® software.

The three-dimensional differences between planned (reference) and placed implants (test) are also illustrated in a color-coded map after setting ±2 mm as accuracy limit. The significance of color code is: green, perfectly matching surface (error ± 0.0995 mm), yellow, test model positively positioned relative to reference, error between +0.0996 and +0.7297 mm, orange, error between +0.7298 and +1.3598, red, error between +1.3599 mm and +2.0000 mm, blue, test model surface negatively positioned relative to reference surface, from – 0.0996 mm (light blue) to −2.0000 mm (dark blue), and gray, test model surface positioned outside the accuracy limit being set (Figure 8).

Immediate or conventional loading of implants was planned according to the CBCT presurgical evaluation and performed after measurements of insertion torque value (ITV) and implant stability quotient (ISQ) with Osstell Mentor® (Gothenburg, Sweden) and the corresponding SmartPeg [21].

Statistical analyses were performed using XLSTAT 2016 (Addinsoft, New York, NY, USA). Mann–Whitney *U* test was used to compare accuracy between maxillary and mandibular surgical templates. A *p* value < .05 was considered significant.

## 3. Results

A total of sixty-five implants were inserted in twenty-five partially edentulous patients: thirty-two in the maxilla and thirty-three in the mandible using tooth-supported surgical templates and a flapless technique. Neither complications nor unexpected events occurred during implants insertion.

Loading protocol was performed as follows: eleven implants were immediately loaded with screw-retained acrylic crowns manufactured prior to surgery, fourty-four implants were early loaded (after 6 weeks' healing period), and ten implants were conventionally loaded due to additional bone graft requirements of the specific sites [22]. No implant was lost at 12 months' follow-up, meaning a 100% survival rate.

The mean length and diameter of the AnyRidge (Megagen Implant, Gyeongbuk, Korea) implants inserted were 9.74 mm (±1.48) and 4.03 mm (±0.40), respectively.

The mean (and standard deviation) of 3D error at the entry point was 0.798 mm (±0.52) and at the implant apex was 1.17 mm (±0.63) and most of the superimposed surfaces were green mapped (error ± 0.0995 mm), indicating a high accuracy level between model (treatment plan) and test (implants placed).

**Figure 9:**
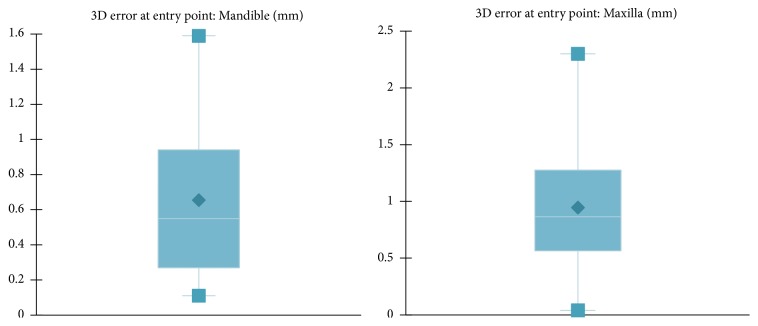
Mean 3D error at entry point, measured at the center of the implant in mandible and maxilla.

**Figure 10:**
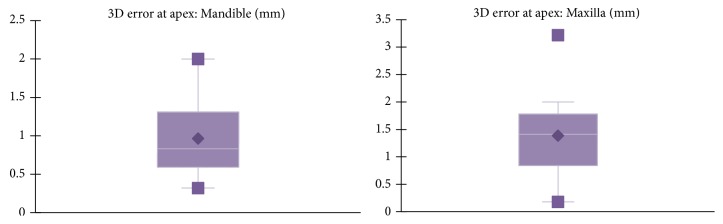
Mean 3D error at the apex measured at the center of the implant in mandible and maxilla.

**Figure 11:**
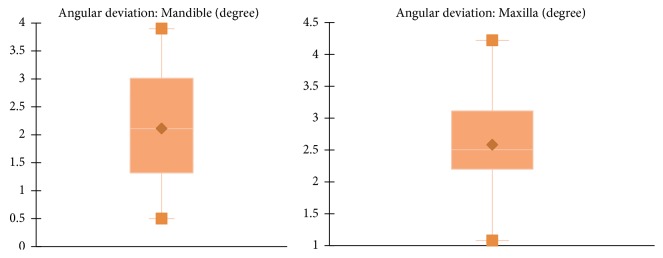
Mean angular deviation for implants inserted in mandible and maxilla.

However, differences in accuracy were noticed when analyzing implants inserted in maxilla and mandible (Figures 9–11 and Table 1). For the mandible, a significantly lower 3D error was observed at entry point *p* = .037, at implant apex *p* = .008, and also in angular deviation *p* = .030 when comparing the 3D error of the implants inserted in the maxilla. No significant difference in accuracy between maxilla and mandible was noticed regarding vertical deviation at entry point (*p* = .314).

## 4. Discussion

To our knowledge, this is the first study assessing in vivo accuracy of computer-guided (static) implant insertion by comparing a digital file (treatment plan) with postinsertion digital impression, without using a postoperative CBCT for this purpose.

The protocol proposed for evaluating planned and performed implants insertion was designed in accordance with the recommendations stated by Bornstein and coworkers [23] regarding the use of new methods such as digital impressions for studies on accuracy of guided implant placement.

In order to compare two virtual objects (treatment plan and scanned implant position), the stl files were imported in Geomagic Qualify software (Rock Hill, SC, USA), recommended as a powerful industrial inspection tool, previously used in dental research to assess conventional impression technique and digital impression [20] and also intraoral and extraoral scanners [24].

The superimposition of the two stl files (treatment plan and digital impression of the implants placed) was performed with point registration, by setting the landmark points on the neighboring teeth. The software then calculated the matrix for the best fit between surfaces (stl files), treatment plan was set as reference, and the locations of the placed implants were compared to the virtually planned implants. A similar superimposition protocol, but for comparing pre- and postoperative CBCT files, was used by Turbush and Turkyilmaz [25] in an in vitro study on acrylic resin mandible for assessing the accuracy of implant placement by using 3 different types of surgical guide: bone-supported, tooth-supported, and mucosa-supported.

The point (or marker) based registration used to match the stl files is considered an accurate and fast method for superimposition, but depending on the number and the location of the markers (remaining teeth) [26]. Therefore, the heterogeneous distribution of the remaining teeth could be considered one of the limitations of the present study.

The performance of computer-guided implant systems and their accuracy relies on all the cumulative and interactive errors involved, from examination, impression, CBCT data acquisition, and guide manufacturing to the surgical procedure and improvements of templates design should be performed to reduce inaccuracy [12].

The aim of this study was to evaluate the accuracy of computer-guided dental implant insertion in partially edentulous patients with the use of a stereolithographic template with sleeve structure incorporated into the design. The drilling system used allowed a higher accuracy of implant placement comparing to the dates recently reported in the literature. From the 65 consecutive implants inserted with the direct drill-guiding system, the placement errors measured were 0.79 (max. 2.30 mm) [27] at the entry point and 1.17 (max. 3.22 mm) at the apex, within the acceptable lower range of error in the literature. Tahmaseb and coworkers [17] in a systematic review analyzing data retrieved from 24 studies reported an inaccuracy at the implant entry point of 1.12 mm with maximum of 4.5 mm on 1,530 implants, respectively, and an inaccuracy of 1.39 mm at the apex of implants with maximum of 7.1 mm when measured on 1,465 implants [17].

The maximum inaccuracy registered (3.22 mm) was measured for 11.5 mm length implant inserted in the posterior maxilla. The length of the implant, the softer bone in maxilla allowing slightly deviation during hand ratchet insertion, and also the limited access with surgical instruments in the posterior area [6] could cause this high placement error.

A significantly better 3D overall positional accuracy was noticed in the mandible comparing to the maxilla, results similar to Ozan et al. [28] findings. Other studies reported no difference [17, 29] or lower accuracy [30, 31] when the guide was used in mandible.

The most notable error with guided surgery was expected to occur in vertical direction (too superficial implant position) due to the presence of debris in the implant cavity [12] or to the blockage of the implant holders in the sleeves of the guide during surgery [32]. However, the use of a guide sleeve incorporated in the design with no need for additional metal sleeves and also the presence of the additional buccal window allowed debris removal during drilling and irrigation results in a reduced vertical deviation 0.50 (±0.38) when compared to Farley and coworkers findings (1.24 mm ± 0.78 mm). The obtained values were also lower comparing to the findings of Lee and coworkers [19] on 21 consecutive implants. The authors reported a 0.925 (±0.376) depth inaccuracy using the same type of surgical template but a different implant (AnyOne, Megagen Implant, Gyeongbuk, Korea), involving different drilling sequences.

The angle deviation value 2.34 (±0.85) from the present study was lower than the mean rate (3.89) reported in the systematic review conducted by Tahmaseb and coworkers [17] but similar to the deviations reported by Lee and coworkers [19] utilizing the same guided implant system. The sleeve incorporated stereolithographic surgical template for flapless implant insertion is designed to lower mechanical tolerance of surgical instruments [19], considered by Vercruyssen and coworkers [12] a source of error occurring during execution phase, leading to improper implant positioning.

Generally, the inaccuracy of the implants insertion expressed by the four parameters recommended being assessed [33]: deviation at the entry point; deviation at the apex; deviation of the long axis (angular deviation); and deviation in height/depth registered in our study lower values than the mean obtained from other studies confirming that the use of shank-modified drills and sleeve incorporated stereolithographic templates is an effective way to improve the accuracy of implant placement.

The results of this study support the rejection of the null hypothesis, both regarding the inaccuracy between planned and inserted implants and also regarding 3D deviation in maxilla and mandible implants.

## 5. Conclusions

The surgical template with sleeve incorporated, designed to reduce mechanical tolerance of surgical instruments, used in the present study has proved high accuracy for dental implants insertion.

By comparing the treatment plan digital file with postinsertion digital impression, without requiring postoperative CBCT for assessing implant placement accuracy, a further radiation exposure may be avoided. However, a validation study comparing error analysis using postoperative CBCT versus intraoral optical scans should be performed in order to evaluate the potential errors arising from impression taking (error of the optical scanner), superimposition of the surfaces, segmentation of implants in the software, error calculation algorithm, and so forth.

Within the limits of the present study, assessment of insertion accuracy by comparing treatment plan stl file and optical impression of implants placed may be considered a promising protocol for guided surgery evaluation in larger prospective clinical trials.

## Figures and Tables

**Table 1:** Discrepancy values at entry point, apex, angular deviation, and vertical deviation.

	Overall (*n* = 65) implants Mean (SD)	Mandible (*n* = 33 implants)			Maxilla (*n* = 32 implants)		
		Mean (SD)	Max.	Min.	Mean (SD)	Max.	Min.
3D error entry point (mm)	0.79 (±0.52)	0.65 (±0.43)	1.59	0.11	0.94 (±0.56)	2.30	0.04
3D error apex (mm)	1.17 (±0.63)	0.96 (±0.49)	2.00	0.32	1.38 (±0.69)	3.22	0.18
Angular deviation (degree)	2.34 (±0.85)	2.11 (±0.88)	3.90	0.50	2.58 (±0.75)	4.22	1.08
Vertical deviation at entry point (*z*-axis, mm)	0.50 (±0.38)	0.46 (±0.34)	1.54	0.00	0.55 (±0.42)	1.96	0.02
